# Early diagnosis of oral submucous fibrosis using salivary 8-OHDG and 8-Isoprostane

**DOI:** 10.6026/9732063002001042

**Published:** 2024-09-30

**Authors:** Bireswar Roy, Shaili Ghosh, SS Mohamed Abdulcader Riyaz, Madhvika Patidar, Urmi Mehta, Priya Agarwal, Pushkar Gupta

**Affiliations:** 1 Department of Oral Pathology and Microbiology, Sudha Rustagi College of Dental Sciences and Research, Faridabad, Haryana, India; 2 Kalinga Institute of Dental Sciences, Bhubaneswar, Odisha, India; 3 Department of Oral and Maxillofacial Diagnostic Sciences, College of Dentistry, Ar Rass Branch, Qassim University, Saudi Arabia; 4 Department of Oral Pathology and Microbiology, Government College of Dentistry, Indore, M.P, India; 5 Dental Surgeon, Gujarat, India; 6 Department of Oral Medicine and Radiology, Modern Dental College and Research Centre, Indore, M.P, India; 7 Department of Prosthodontics and Crown & Bridge, Hitkarini Dental College and Hospital, Jabalpur, M.P, India

**Keywords:** DNA damage, gutka chewing oxidative stress, OSMF, saliva, 8-OHdG, 8-Isoprostane

## Abstract

Oral submucous fibrosis (OSMF) is a condition that may be cancerous. The prognosis of OSMF is determined by a number of biomarkers, including 8-hydroxy 2' de-oxy-guanosine and 8-isoprostane. It is possible to assess the levels of 8-OHdG and 8-isoprostane in blood and saliva. Therefore, it is of interest to estimate salivary 8-OHdG and 8-isoprostane levels in order to diagnose oral submucous fibrosis. A sample size of 40 was divided into two groups with 20 samples in each, *i.e.*, Group I - Healthy group (gutka consumers without any lesion) and Group II -Test (gutka consumers with OSMF). Samples of serum and saliva were taken from each group. Then samples were centrifuged for 15-20 minutes at 1000 RPM and 2-8°C. The resulting supernatant was pipetted out into labelled Eppendorf tubes in a volume of 1.5 ml, and it was then kept at 80°C. 8-OHdG and 8-isoprostane concentrations in various samples were determined using the ELISA technique. Serum's 8-OHdG content was considerably lower than saliva sample (P-value <0.05). The test group exhibited increased concentrations of 8-OHdG and 8-isoprostane in both saliva and serum samples when compared to the control group. 8-OHdG and 8-isoprostane can be utilised to diagnoses of OSMF.

## Background:

Oral submucous fibrosis (OSMF) is a potentially fatal condition that is more common in India than in other Asian nations [1, 2]. The main symptoms include blanching of the mucosa, burning when eating spicy food, and difficulty in opening the mouth because of fibrous bands [3]. It is defined as an inflammatory disorder of the oral mucosa linked to an excess of oxidants and a deficiency of antioxidants. The underlying cause of these oral problems is juxta-epithelial inflammatory reactions that result in fibro-elastic alterations of the lamina propria and, in turn, in the oral mucosa becoming stiff [4]. 7%-13% of OSMF cases progress to oral squamous cell carcinoma (OSCC) due to malignant transformation [5]. One of the most frequent causes of OSMF, which can then lead to the malignant transmission of OSMF, is gutka eating. Several carcinogenic components, including tobacco, betel nut, limestone, catechu, and crusted glass, are added to make gutka. These gutka ingredients aid in the generation of several free radicals, including OH2, O2, RO, ROOH, ROO, and H2O2. One of the possible causes of DNA damage is radiolysis of water, which produces the OH ion. Because DNA is susceptible to both endogenous and external damage, it is chemically unstable and prone to oxidation [2, 6]. The term "oxidative stress" refers to the situation in which a cell's oxidation surpasses the body's antioxidant repair mechanisms and is used to explain the relationship between free radicals and disease. The extremely erratic and volatile Reactive oxygen species (ROS) cause oxidative damage, which in turn modifies the bases of deoxyribonucleic acid (DNA) [7]. The generation of oxygen-free radicals, namely hydroxyl radicals (HO-), has the potential to seriously damage DNA strands [8]. One of the most dangerous oxygen-free radicals that can harm essential biomolecules such membrane lipids, cellular proteins, and DNA is the hydroxyl radical (HO-)[7]. Mutagenesis and carcinogenic processes are caused by DNA damage [2].Precancerous conditions, cancer and its prognosis are diagnosed using biomarkers. In recent years biological characteristics, polymorphism, promoter methylation, microRNA, mRNAs, non-coding RNAs and protein and trace elements in a solid biopsy, liquid biopsy from serum, and saliva have all been employed as possible biomarkers for OSMF. Samples from saliva, serum, tissue and cytology can all be used for analysis with special benefits [9]. A number of biomarkers, including 8-hydroxy 2' deoxyguanosine, 8-isoprostane, malondialdehyde (MDA), exhaled volatile alkanes, S100A7 and lipid hydroperoxides, are utilised to determine the prognosis of OSMF. A frequent indicator of oxidative stress and the antioxidant status in cancer patients is the quantity of Malondialdehyde [1-2]. 8-hydroxy 2' deoxyguanosine, often known as 8 OHdG, has emerged as a biomarker of oxidative stress in bodily fluids and tissues in recent years. The most frequent stable byproduct of free radical-induced oxidative DNA damage is 8-OHdG. The molecule's eighth position can be changed with a hydroxyl radical to generate the guanine-modified product 8 OHdG [10]. The most common and mutagenic lesion in nuclear DNA is oxidatively modified DNA (8 OHdG), which is significant in the processes of mutagenesis and carcinogenesis and may be measured to show the degree of damage to genetic material [11]. The primary reason 8 OHdG is a commonly utilised biomarker for oxidatively induced DNA damage is that it can be reliably detected. It has been shown that in a number of cancer situations, patients had elevated levels of 8 OHdG when compared to healthy persons [2]. A comparatively non-invasive, easy-to-use, and effective approach for tracking oxidative stress in patients with premalignant oral diseases is the characterisation of 8-OHdG from saliva samples [7]. These days, 8-isoprostane is becoming an important oxidative stress marker. Its chemical stability gives it an advantage over other oxidative stress markers. 8-isoprostane is employed as a putative marker for OSF and oral cancer [1, 12]. It is possible to identify biomarkers in tissue, blood, urine and saliva. In place of blood- and serum-based diagnostic methods, saliva-based diagnostics are non-invasive, non-infectious, and provide affordable screening tools [13]. Therefore, it is of interest to estimate the levels of salivary 8-OHdG and 8-Isoprostane as a diagnostic marker for oral submucous fibrosis.

## Materials and Method:

This study was conducted in the Department of Oral Maxillofacial Pathology and Oral Microbiology. The study was done after attaining the approval from institutional ethics committee and informed consent from participants. A total sample size of 40 was divided into two groups with 20 samples in each, *i.e.*, Group I - Healthy group (gutka consumers without any lesion) and Group II - Test (gutka consumers with OSMF). Samples of serum and saliva were taken from each group. Using drooling techniques, about 5 cc of unstimulated saliva were obtained in the morning. A 20 gauge needle was used to aspirate 5 millilitres of blood. After that, samples were centrifuged for 15 to 20 minutes at 1000 RPM and 2 to 8°C. The resulting supernatant was pipetted out into labelled Eppendorf tubes in a volume of 5 ml, and it was then kept at 80°C. The ELISA test was used to determine the concentration of 8 OHdG protein in various samples at 450 nm after adding stop solution in a 96-well microplate using 1.5 ml of blood and 2.5 ml of saliva. Likewise, 2.5 ml of saliva and 1.5 ml of blood samples were exposed to the 8-Isoprostane ELISA technique. SPSS software version 23.0 was used to statistically evaluate the collected data using the t test and post hoc test with p<0.05.

## Results:

The patients ranged in age from 22 to 52 years old, with a mean age of 32.1 years. In both groups, salivary samples had a greater 8-OHdG concentration than serum samples, suggesting that salivary 8-OHdG is a reliable predictor. There was a substantial (P<0.001) intergroup difference in the concentration of 8-OHdG in both serum and saliva (Table 1). For both saliva and serum samples, the test group's 8-isoprostane concentration was higher than that of the control group (Table 2). P<0.001 indicates that the difference is statistically considerable.

## Discussion:

Oral submucous fibrosis (OSMF) is classified as a potentially malignant condition that primarily affects Indians who chew areca nut [3]. In cells, oxidative stress triggers a number of detrimental biochemical processes that affect DNA, lipids, proteins, and cellular membranes. 8-OHdG is eliminated from cells via the ATP-dependent active cellular transport mechanism and passively absorbed into the bloodstream. 8-OHdG can appear as either free 8 OHdG or 8-OHdG integrated into DNA. The different distributions of 8-OHdG protein in serum, urine, and saliva suggest that the oxidative stressors caused the creation of 8-OHdG protein by DNA oxidation [2]. An imbalance between antioxidants and reactive oxygen species (ROS) leads to oxidative stress, which is a major factor in oral cancers including oral squamous cell carcinoma (OSCC). 8-isoprostane, have been used as disease indicators in tissue fibrosis and other disorders [14]. Prajapati *et al.* assessed individuals with oral submucous fibrosis for serum, urine, and salivary 8-OHdG. They came to the conclusion that, when compared to serum and urine, saliva seems to be the best suitable sample type for evaluating 8 OHdG in OSMF participants [2]. Salivary 8-hydroxydeoxyguanosine (8-OHdG) levels were measured by Nandakumar *et al.* as a possible DNA Damage biomarker in OSMF. They came to the conclusion that 8-OHdG can be employed as a unique DNA damage biomarker to gauge the course of a disease [7]. The systematic meta-analysis by Alarcón-Sánchez *et al.* revealed elevated levels of 8-OHdG in the saliva of patients with oral cancer [8]. Our findings are consistent with these results. In individuals with OSMF, Kulasekaran *et al.* found 8-OHdG expression and compared it to both normal buccal mucosa and various OSMF grades. They came to the conclusion that there is a statistically significant variation in the levels of 8-OHdG expression among the research groups [15]. Kaur *et al.* used salivary 8-hydroxy-2-deoxyguanosine (8-OHdG) and malondialdehyde (MDA) to examine oxidative DNA and lipid damage. According to this study, precancerous and squamous cell carcinoma (SCC) individuals had oxidative DNA and lipid damage [13]. Increased levels of oxidative stress indicators, such as MDA, 8-hydroxy-2'-deoxyguanosine and salivary 8-isoprostane, were detected in the saliva of OSMF patients, according to Saso *et al.*'s systematic review [4]. The normal OSMF had an average 8-isoprostane level in saliva of 3.2 ng/mL, while the normal OSMF had an average of 5.5 ng/mL. In comparison to control groups, Meera *et al.* discovered that OSMF cases had greater levels of 8-isoprostane [1]. This is consistent with what we discovered. The number of studies that have used saliva as a biological sample for oral malignancies has expanded significantly in recent years since it is a readily collected medium and can be evaluated for tumour markers because it is in contact with the lesion. We discovered that 8 OHdG and 8-isoprostane can be utilised to determine the prognosis of OSMF.

## Conclusion:

Saliva and plasma in OSMF contained detectable amounts of isoprostane. Compared to serum, saliva seems to be the most suitable sample type for assessing 8 OHdG in OSMF patients. Two potential biomarkers for OSMF diagnosis can be 8-isoprostane and 8 OHdG.

## Financial support and sponsorship:

Nil

## Competing Interests:

None

## Figures and Tables

**Table 1:** Comparison of 8-OHdG in saliva and serum for healthy and control group

**Group**	**Mean±SD (ng/mL)**		**F**	**P**
	**Saliva**	**Serum**		
Group I- Healthy (Control)	1.7275±0.24689	0.3254±0.11367	23	0
Group II- Test (OSMF)	1.7834±0.22468	0.3684±0.14385	6.3	0
*p*	0.001	0.001		

**Table 2:** Comparison of 8-Isoprostane in saliva and serum for healthy and control group

	**Mean±SD(ng/mL)**	
	**Saliva**	**Serum**
Group I- Healthy (Control)	3.093±0.124	284.367±0.1365
Group II- Test (OSMF)	5.246±0.325	328.536±0.3256
F	18.6	5.28
*p*	0.001	0.001
